# The mechanobiology of nuclear phase separation

**DOI:** 10.1063/5.0083286

**Published:** 2022-04-28

**Authors:** Daniel S. W. Lee, Amy R. Strom, Clifford P. Brangwynne

**Affiliations:** 1 Lewis-Sigler Institute for Integrative Genomics, Princeton University, Princeton, New Jersey 08544, USA; 2 Department of Chemical and Biological Engineering, Princeton University, Princeton, New Jersey 08544, USA; 3 Princeton Institute for the Science and Technology of Materials, Princeton University, Princeton, New Jersey 08544, USA; 4 Howard Hughes Medical Institute, Princeton University, Princeton, New Jersey 08544, USA

## Abstract

The cell nucleus can be thought of as a complex, dynamic, living material, which functions to organize and protect the genome and coordinate gene expression. These functions are achieved via intricate mechanical and biochemical interactions among its myriad components, including the nuclear lamina, nuclear bodies, and the chromatin itself. While the biophysical organization of the nuclear lamina and chromatin have been thoroughly studied, the concept that liquid–liquid phase separation and related phase transitions play a role in establishing nuclear structure has emerged only recently. Phase transitions are likely to be intimately coupled to the mechanobiology of structural elements in the nucleus, but their interplay with one another is still not understood. Here, we review recent developments on the role of phase separation and mechanics in nuclear organization and discuss the functional implications in cell physiology and disease states.

## I. INTRODUCTION

**FIG. 1.**
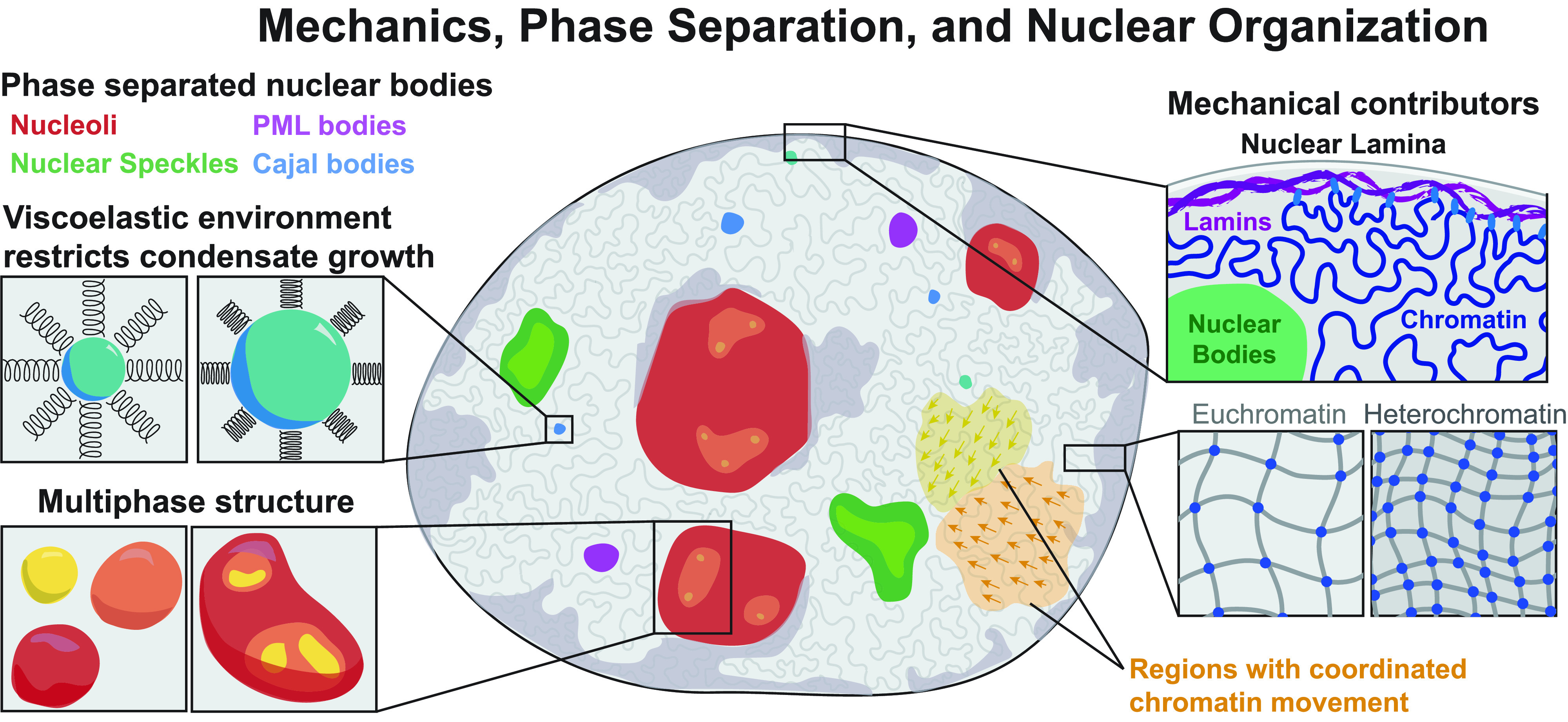
The nucleus is host to a wide array of liquid-like nuclear condensates, e.g., nucleoli, PML bodies, nuclear speckles, and Cajal bodies, which form by phase separation, and is also largely filled with chromatin, which is highly structured and exhibits dynamic micrometer-scale correlations. Chromatin is a viscoelastic material with heterogeneous pore sizes and structure throughout the nuclear environment, and liquid-like condensates deform the chromatin matrix as they grow. These components directly interact in multiple contexts and elucidating the physical interplay between phase separation and nuclear mechanics is critical for understanding nuclear organization.

The nucleus is a large membrane-bound organelle that organizes the genetic material of the cell, serving to maintain genomic integrity while also orchestrating gene expression. In order to coordinate these activities, the nucleus is composed of multiple functionally and spatially distinct structures which together impact its collective material properties. The biological macromolecules that makeup these structures dictate their mechanics, which has been well-studied in the context of the lamina, the structural scaffold supporting the nuclear envelope, and chromatin, the material into which DNA and associated proteins are packaged (Fig. 1). The lamina is structurally composed of a meshwork of intermediate filaments of type A lamins (lamin A and C) and type B lamins (lamin B1 and B2/B3), as well as membrane-associated proteins, which support the inner nuclear membrane and tether chromatin to the nuclear periphery.^1^ Interphase chromatin is a heterogeneously compacted polymer crosslinked by transiently binding proteins^2^ that constitutes a large volume fraction of the nucleus. The nucleus exhibits bulk viscoelasticity, which is modulated by the structure of both the lamina and the chromatin, and can vary depending on cell fate.^3–7^ These advances have been foundational to our understanding of the functional role of nuclear mechanics, but they have largely ignored the mechanical impact on and interplay with a third primary constituent of the nucleus: the membraneless nuclear condensates.

Nuclear condensates have been observed for many decades and well over a century in the case of nucleoli and Cajal bodies.^8,9^ However, until recently, our understanding of these structures was largely qualitative and descriptive. A detailed catalog of the molecular players for some of these structures has now been uncovered, together with information about their interactions and functional importance. Still, we lacked a predictive framework for understanding how these myriad interactions give rise to emergent collective organization—the coherent structures that are clearly more than the sum of their parts.

Liquid–liquid phase separation (LLPS) has recently been appreciated as an important organizing principle for the cell, resulting in the formation of membraneless biomolecular condensates that function as organizational hubs, sequestration centers, or reaction crucibles.^10^ Within the nucleus, numerous diverse aspects of gene expression appear to be facilitated by a litany of these condensates (Fig. 1). These include nucleoli,^11^ promyelocytic leukemia (PML) bodies,^12^ nuclear speckles,^13^ and Cajal bodies^14^ as well as repressive heterochromatin foci^15,16^ and the enigmatic superenhancer.^17–19^

The concept that phase transitions underlie the self-organization of non-living matter is at the core of engineering and the physical sciences, rooted in statistical thermodynamics. This provides a well-established quantitative and conceptual framework, which is being productively adapted to describe various aspects of cytoplasmic and nuclear condensate organization. The role of phase transitions in cell biology has been described in detail in several excellent reviews.^10,20,21^ Here, we focus on recent progress toward understanding the mechanical interplay between phase separated nuclear condensates with other nuclear structures. First, we provide a broad overview of the principles underlying the assembly of nuclear structures, considering relevant theoretical concepts and then using this framework to contextualize recent experimental advances. We then consider future work required for a unified model of nuclear organization and finally discuss evidence for the biological control and mechanochemical feedback on condensates.

## II. NUCLEAR ORGANIZATION AND MECHANICS

**FIG. 2.**
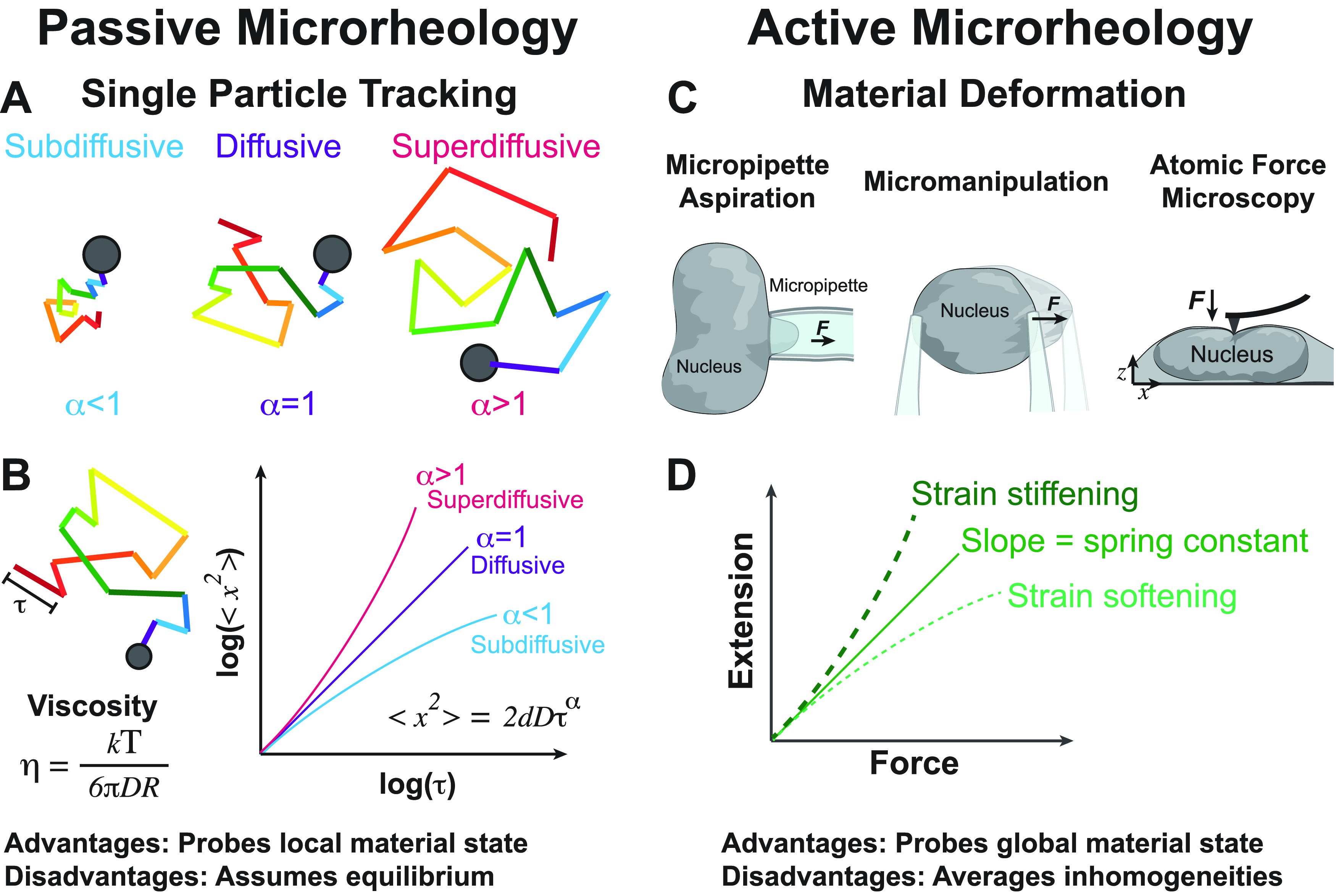
Viscoelasticity in the nucleus can be interrogated by a suite of methods known as microrheology. In passive microrheology (left column), tracer particles are tracked over time (a) and their movement is quantified by calculating a mean squared displacement (b), which typically scales as a power law in the time lag with exponent α. α = 1 indicates a purely viscous solution, while subdiffusion, i.e., α <1, which is typical of structures in the nucleus, is indicative of viscoelastic constraints, and α >1 is indicative of driven, active motion; these behaviors reflect local activity and mechanics, e.g., of chromatin. In active microrheology, a calibrated force is applied to the material and the deformation is used to infer the material's mechanical properties. For the nucleus, this is typically used to extract bulk mechanics via methods such as micropipette aspiration and atomic force microscopy (c). Typically, a force-extension curve is calculated, whose slope in the linear or elastic regime corresponds to the spring constant for displacements; nonlinearity indicates that the material is strain-stiffening or strain-softening (d). These techniques have defined the mechanical contributions of particular biological components of the nucleus such as the lamina and chromatin.

The mechanical properties of the nucleus are functionally relevant both to protect the genome from external mechanical insult and to coordinate the internal interactions necessary for various processes, including transcription and DNA repair. The majority of the interior of the nucleus is composed of chromatin. Electron microscopy studies suggest that chromatin forms a disordered chain of 5–24 nm in diameter that fills the nucleus with a strikingly high volume fraction during interphase,^22^ ranging from 15% to 65% dependent on local compaction. Spatial genomic mapping techniques including Hi-C^23^ have demonstrated three-dimensional folding patterns of the linear chromatin polymer. However, these studies do not address the bulk material properties of the nucleus, which emerge from this underlying structural organization. In particular, much remains unclear about the relationship between higher-order biomolecular organization within the nucleus and bulk properties such as viscoelasticity, which is thought to be critical for biological functions including differentiation and mechanosensation. For example, nuclei of cells grown in stiffer environments take on stiffer rheological properties,^7^ which are associated with differential gene expression profiles.^24^ Moreover, the presence of highly viscous, mesoscopic condensates should impact both the local and global mechanical properties of the nucleus. However, these properties can only be elucidated by performing dynamic measurements in living cells (Fig. 2). In general, viscoelasticity can be measured by a suite of techniques and approaches known as (micro)rheology, which has active and passive flavors.^25–27^

Passive microrheology is performed by tracking the trajectories of tracer particles embedded within the material of interest [Fig. 2(a)]. The fluctuating motion of the particle trajectories are then analyzed by calculating the mean squared displacement (MSD), which often exhibits power law scaling with time lag τ and exponent α: $\langle x^{2}\rangle =2dD\tau^{\alpha }$, where *d* is the dimensionality of the system and D is the diffusion coefficient [Fig. 2(b)]. An exponent of α=1 is termed diffusive and is consistent with particles performing a random walk through a purely viscous liquid medium. For such a simple liquid in equilibrium, the diffusion coefficient can be used to extract the fluid viscosity η, through a form of the fluctuation–dissipation relation known as the Stokes–Einstein equation $\eta =\frac{kT}{6\pi DR}$, where kT is the thermal energy scale given by the product of Boltzmann's constant k and the absolute temperature T, and R is the particle radius;^28^ more viscous fluids exhibit less dynamic Brownian motion (smaller D). Particles that move according to an exponent α<1 are termed subdiffusive, which is generally an indication of viscoelasticity, while those with α>1 are described as superdiffusive, which is associated with active, driven motion.

While the underlying conceptual framework of passive microrheology is clear and well-understood, experimentally introducing inert particles into living cells can be challenging and low throughput. In some cases, cells can be induced to express genetically encoded tracer particles, such as genetically encoded multimeric nanoparticles (GEMs), whose motion can be tracked and analyzed.^29,30^ Alternatively, experimentalists commonly track the motion of endogenous structures. Often, however, objects that might be tracked are small and/or too dense, such as with fluorescently labeled histones. In such cases, rather than calculating discrete trajectories for each particle, “continuum” image analysis methods can be used, such as displacement correlation spectroscopy (DCS),^31^ or particle image velocimetry (PIV), which extracts locally averaged displacement fields rather than individual particle trajectories, expanding the utility of passive microrheological techniques beyond the scope of discrete beads or organelles.

Despite the power of these approaches, passive microrheology generally requires that the material being studied is homogeneous and in equilibrium, which is often problematic in living cells, due to their strong heterogeneity and out-of-equilibrium activity. In principle, heterogeneity in soft materials can be dealt with using approaches such as two-point microrheology,^32^ although it has rarely been successfully deployed in living systems, in part due to the out-of-equilibrium activity ubiquitous in cells. For example, in human cells, it has been shown that chromatin exhibits adenosine triphosphate (ATP)-dependent, micron-scale dynamic correlations.^31^ Similarly, chromatin displacement has been shown to be highly heterogeneous, with particle image velocimetry suggesting that the local viscoelasticity of the nucleus varies by compartment.^33^ Despite these confounding aspects of a typical cell nuclei, the *Xenopus laevis* oocyte provides a simpler, contrasting example. Mature *X. laevis* oocytes are ∼1 mm in diameter and relatively homogeneous, featuring a low chromatin volume fraction. Analysis of the MSD of beads injected into oocyte nucleoplasm compared with “active” microrheological measurements of beads sedimenting through the nucleoplasm under gravity, suggest that this particular system is not strongly out of equilibrium.^34^

While the fluctuation–dissipation theorem may only sometimes hold in cells, precluding the precise determination of material parameters, tracking injected beads or endogenous structures can still shed light on apparent material state and biological activity impacting transport dynamics within the cytoplasm and nucleus.^26^ For instance, individual genomic loci, such as telomeres,^35,36^ exhibit strong subdiffusive motion in multiple systems,^37,38^ implying the existence of viscoelastic constraints on their motion. Interestingly, telomeres recover normal diffusion following lamin A/C knockdown,^35^ suggesting that subdiffusion may be due to the mechanical structure of the nucleus.

In addition to suggesting that the chromatin network is highly viscoelastic, subdiffusive motion of nuclear components has been the subject of much experimental and theoretical study due to the relevance of the “first passage time” or genomic search problem.^39,40^ Inside the crowded nucleus, critical processes may be limited by the time it takes for two loci to diffuse into proximal locations, including enhancer–promoter interactions^41–44^ and homology search during homology-directed repair of DNA double-stranded breaks.^39,40,45^

In addition, recent work has suggested that chromatin may lie near a sol–gel transition to facilitate a transition to rapid, liquid-like dynamics when functionally required^46^ and that DNA damage may drive directional motion of chromatin.^47,48^ The presence of liquid-like nuclear condensates may facilitate this genomic search, and hence transcription, by providing an environment with rapid, liquid-like dynamics, which have been observed coupled to transcription^49^ or excluding nonspecific bulk chromatin.^50^ Together, this evidence may suggest that condensates play an important role in spatially organizing chromatin and regulating the viscoelastic properties of the local environment of the nucleus, although their role in the maintenance of bulk material properties remains unclear and a topic for future study.

Bulk material properties are more easily measured using active microrheological approaches, which do not rely on assumptions of thermodynamic equilibrium, and provide a direct means of interrogating the bulk material properties of the nucleus and subnuclear structures; these approaches have been reviewed in depth elsewhere.^51^ Briefly, these approaches utilize force directly applied to microscopic materials and measurement of the resulting displacement [Fig. 2(c)]. The calculated force-extension curve, which is analogous to the stress–strain curve common in materials science, is generally linear over small displacements, with the fit slope representing a spring constant for the material. For larger displacements, the response is often non-linear, a behavior termed either strain-stiffening or strain-softening if the response curve is, respectively, concave up or down [Fig. 2(d)]. Various active microrheology techniques, utilizing tools such as magnetic or optical tweezers, atomic force microscopy, or micropipette aspiration, have been applied successfully to show that chromatin viscoelasticity is a major determinant of the bulk mechanical properties of the nucleus.^3,52–54^ However, one crucial pitfall of these techniques is that they average out spatial heterogeneity and substructure. In order to assay properties with more granularity, active microrheological methods must be combined with targeted genetic or pharmacological perturbations. For instance, micromanipulation has revealed that bulk stiffness of isolated nuclei under small deformations is determined by chromatin, which is influenced by epigenetic modifications,^4,55^ the chemical “tags” the cell uses to alter gene expression. In contrast, for larger deformations, the response is dominated by the lamin shell at the nuclear periphery. Genetic knockdown of lamin proteins lowers long-extension regime stiffness, while tuning chromatin compaction state via epigenetic modifications alters short-extension regime stiffness.^4^

Because organization of heterochromatin, the more compacted, stiffer form of chromatin, is thought to occur in part through LLPS of proteins and DNA,^15,16,56,57^ LLPS may have a direct role in determining bulk nuclear mechanics. Indeed, auxin-induced degradation of a key heterochromatic organizational protein, HP1α, results in softer nuclei measured by micromanipulation.^58^ HP1α is also capable of mechanically compacting DNA *in vitro*^59^ without the presence of ATP, which may underlie the dependence of bulk nuclear mechanics on HP1α protein level.^58^ Finally, large and viscous nuclear condensates like nucleoli may also contribute to bulk nuclear mechanics measurable by active microrheology, potentially through structural reorganization of heterochromatin and/or more open euchromatin, as discussed further below. Overall, these passive and active microrheological tools have provided a foundational framework upon which to build our understanding of nuclear mechanics and will increasingly be deployed to elucidate mechanical links to LLPS.

## III. PHASE SEPARATION IN NUCLEAR ORGANIZATION

Membraneless nuclear condensates, including the nucleolus, are formed via phase separation within the chromatin-rich nuclear environment.^60^ In addition to its impact on the bulk mechanics of the nucleus, the viscoelasticity of chromatin also impacts the formation of these condensates. Condensates typically form in chromatin-poor regions,^50,61^ suggesting that phase separation occurs more readily in softer environments, as has been observed for *in vitro* systems.^62^ While typical equilibrium phase separation would predict that condensates would coarsen into a single large body over time, phase-separated nuclear bodies tend to have characteristic size and number,^63^ which can be significantly altered in disease.^64^

It was recently proposed that the cell can regulate number, size, and location of nuclear condensates by utilizing the general elastic constraints of the crowded nucleus.^65^ In this study, chromatin-excluding engineered condensates were used as microrheological probes of nuclear mechanics, exhibiting MSDs similar to tracked loci. Supporting this hypothesis, growth of nucleoli has similarly been shown to be constrained by the presence of a nuclear actin scaffold in *X. laevis* oocytes, sedimenting and coalescing into a single massive nucleolus after actin disruption.^11,34^

The kinetic arrest of nuclear condensate coarsening due to viscoelastic chromatin constraints suggests the possibility that cells may actively regulate chromatin viscoelasticity to control number and size of nuclear bodies. Interestingly, growth of synthetic condensates mechanically creates a chromatin-poor cavity,^50,65^ demonstrating that phase separation into protein-rich condensates can directly impact chromatin structure. A unified model of nuclear organization will need to consider the interplay between chromatin and phase-separated bodies, but a deeper understanding of the underlying physics is needed. Here, we review phase separation models and recent work done to extend them to the complex intracellular milieu.

### A. Regular solution-based frameworks for biomolecular phase separation

**FIG. 3.**
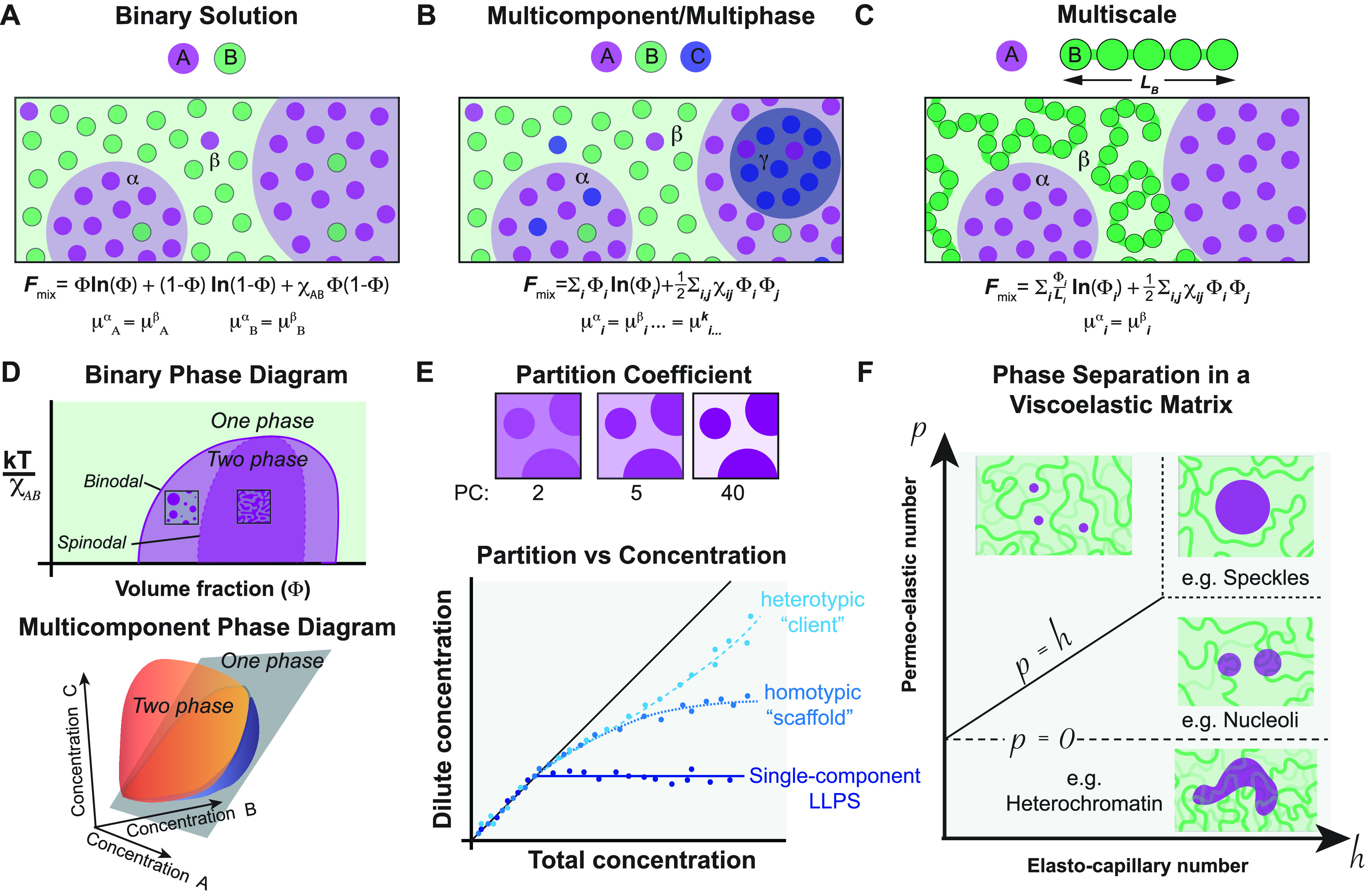
The thermodynamics of phase separation are traditionally understood using models of increasing complexity, building from a simple two-component “regular solution” colloidal system (a), to a multicomponent/multiphase system (b), and finally, to a polymeric system (c) containing components of differing lengths. These systems are typically understood by mapping phase diagrams with two or more components (d), or by calculating partition coefficients (e) in the case of multicomponent systems with intractably complex phase diagrams. Recent theoretical work has suggested that a local viscoelastic matrix can influence the equilibrium size, shape, and morphology of droplets, as a function of the ability of the droplet to wet the matrix and the mechanical stiffness of the matrix, quantified as the permeo-elastic number, *p,* and the elasto-capillary number, h, respectively (f).

In recent years, the utilization of phase separation models drawing inspiration from materials science and condensed matter physics has yielded significant insight into biological organization. In general, phase separation occurs in a solution with two or more components when the energetic favorability of particular intermolecular interactions exceeds the entropic cost of demixing, resulting in a transition from a well-mixed unitary liquid into two physically distinct liquids [Figs. 3(a) and 3(d)]. The simplest theoretical framework is the regular solution model, a mean-field approach that describes the free energy of mixing $F_{\mathrm{mix}}$ of an ideal solution with species A and B [Fig. 3(a)], resulting in a phase diagram where demixing depends on temperature, concentration, and interaction strength (encoded in the interaction parameter $\chi_{AB}$) [Fig. 3(d)]. When $F_{\mathrm{mix}}$ has two local minima, the solution can undergo phase separation, within the “two phase” region of a phase diagram. This can also be described using the chemical potential μ, which is defined as the energy required to add an additional molecule of a particular component i to a mixture with concentration ϕ, i.e., $\mu (\phi )=\frac{\partial F}{\partial N_{i}}$. If two distinct concentrations $\phi_{i}^{\alpha }$ and $\phi_{i}^{\beta }$ exist such that $\mu \left(\phi_{i}^{\alpha }\right)=\mu (\phi_{i}^{\beta })$, then phase coexistence is favorable.^66^ It should be noted that for a two-phase system, the partition coefficient, defined as the ratio of dense to dilute concentration, i.e., ${\phi_{i}^{\beta }/\phi }_{i}^{\alpha }$ is independent of concentration [single component LLPS, Fig. 3(e)].

Despite the attractive simplicity of the regular solution model, living systems exhibit much more complexity than can be fully captured by this framework. For example, in nearly all biological situations, the system will be multicomponent, i.e., having N>2 species. One conceptual simple idea is that only some of these components have particular importance in stabilizing phase coexistence, which have been termed “scaffolds,” while nonessential components recruited to condensates have been referred to as “clients.”^12^ More generally, there is a continuum of relative impact on phase separation, which can be viewed as a multidimensional phase diagram [Fig. 3(d)]. Indeed, the regular solution equations are generalizable to higher dimensions [Fig. 3(b)], although the partition coefficient will no longer necessarily be constant upon varying the concentration of any single component, and instead scale to reflect heterotypic stabilization or destabilization of the phase by a particular component [Fig. 3(e)]. This effect has recently been described for condensates including the nucleolus, Cajal bodies, and stress granules,^67^ providing a set of tools to interrogate the composition-dependent dynamics of multiphase endogenous condensates.

An additional consideration is that large numbers of interacting components can give rise to many distinct coexisting liquids, which is referred to generally as a multiphase system^68–70^ [Fig. 3(b)]. Instances of multiphase behavior wherein distinct phases are in physical contact have been observed in several intracellular contexts, including the nucleolus,^71^ stress granule/P- bodies,^72^ Cajal bodies,^73^ and nuclear speckles.^74,75^ Precise quantitative links to theory are limited, but one approach considers *n* components, whose pairwise interactions are described by a symmetric n×n matrix $\chi_{ij},$ where phase coexistence conditions are expected to be generalized as, i.e., ${\mu_{k\;}}^{i}={\mu_{k\;}}^{j}$ for all phases i≠j and each component *k* [Fig. 3(b)]. Such multiphase structures have been theoretically shown to be a likely stable state.^70^ Taken together, these generalizations build toward a single quantitative framework that enables predictive thermodynamic modeling of multiphase and multicomponent coexistence for complex biomolecular mixtures.

### B. Models of increasing complexity describe phase separation *in vivo*

While the regular solution framework is conceptually powerful, it only considers the interactions of coarse-grained and similar-sized components in the context of “mean field” interactions. However, intracellular phase separation involves polymeric biological macromolecules which have complex properties in their own right. Indeed, a growing body of experimental and theoretical work highlights the rich way in which biomolecular sequence patterning (i.e., protein, RNA, DNA) gives rise to multivalent interaction elements that can exhibit rich phase behavior.^76–79^ However, there are many key unanswered questions about how biopolymer sequences and interactions impact phase separation, in particular, the role of the local mechanical environment, which itself emerges from the interactions and patterning of the cell's myriad polymeric components.

Consider first the simplest way in which the polymeric nature of biomolecular components impacts phase separation. In the case of a polymeric component in solution undergoing phase separation, the very different size between the polymer and solvent necessitates another extension of the regular solution model known as Flory–Huggins theory;^80,81^ in this context, the entropy of mixing is mitigated by limited conformations achievable for a polymer, and for a particular component is multiplied by a factor of $\frac{1}{L}$ for a polymer containing L monomers [Fig. 3(c)]. The length-dependent modification of the Flory–Huggins free energy provides insight into the assembly of multivalent RNA-dependent condensates such as nucleoli as well as the role of oligomerization domains in driving phase separation. Future work will elucidate how these components, in the context of ribosome assembly, may be actively modified inside a phase-separated condensate.^82^

Paralleling the co-condensation of RNA-binding proteins with large RNAs, there is also strong evidence that multivalency associated with DNA plays a central role in phase separation. For example, it has recently been suggested that repetitive sequence motifs in telomeric DNA result in the condensation of Shelterin protein complex components at the repetitive ends of chromosomes, known as telomeres, to preserve genome stability.^36,83^ Similar repetitive DNA motifs could also underlie transcription factor condensation at enhancers.^84^ Moreover, clusters of methylated nucleosomes in heterochromatic regions act as binding platforms for dimers of the phase-separating protein Heterochromatin Protein 1α (HP1α),^85^ which underlies the structure of heterochromatic domain formation.^15,16^ DNA- or RNA-scaffolded phase separation may also be critical for localizing enzymatic modifications like transcriptional repression: phase separation of the polycomb complex PRC1 onto H3K27me3 epigenetic marks is capable of writing H2AK119Ub marks, which are required for repression,^86^ suggesting an active role for phase separation in the regulation of heterochromatic enzymes.^56^ Finally, histones have been shown to phase separate in the presence of DNA *in vitro*, suggesting a broad role for phase separation in chromatin organization.^87^

While there has been significant experimental evidence for phase separation associated with DNA scaffolds, theoretical work has been lacking. Building from decades of work in polymer physics, traditionally applied to synthetic polymers,^88^ Flory–Huggins theory has previously been applied in the context of DNA compaction by DNA-binding proteins.^89^ Consistent with fundamental polymer physics, DNA can be considered a scaffold component of a phase separating system,^90^ despite some distinctions made in the literature between “liquid–liquid” and “polymer–polymer” phase separation.^91^ However, the mesoscale stability of these domains is generally poorly understood, as the dynamics of coarsening and nucleation will tend to be suppressed by the presence of a less dynamic scaffold.

Future work will examine the ability of phase-separated droplets to compact and confer rigidity to DNA in living cells, the role of nonequilibrium activity (e.g., epigenetic readers and writers), and how the properties of the condensates themselves are regulated.

### C. Nuclear mechanics and the dynamics of phase-separated condensates

As discussed above, the typical prediction for the most energetically favorable state of a condensate is a single large body, but condensates typically form in numerous locations at once, and mechanical constraints within the nucleus can provide kinetic barriers to slow the subsequent coarsening process. Addressing the question of where and when multiple droplets initially form, and the potential for the complex intracellular environment to similarly impact this process, requires a quantitative description of the earliest stages of phase separation.

In general, droplets first form by one of two mechanisms: nucleation followed by growth or spinodal decomposition. Because the latter generally requires a quench into high concentrations deep within the two-phase region [Fig. 3(d)], in the context of biomolecular phase separation it has only been reported in engineered systems^90^ and *in vitro.*^92,93^ If the system exists in the binodal region closer to the phase boundary, droplets will form by a process known as nucleation and growth. In this case, the system is metastable, and droplet formation must overcome an energetic barrier associated with surface tension, γ, and chemical potential, Δμ, occurring at a rate related to the magnitude of the energy barrier according to classical nucleation theory.^94^ Since the destabilizing surface tension scales with surface area, but the stabilizing thermodynamic drive to phase separate (i.e., chemical potential) scales with volume, phase separation is only favorable above a characteristic size known as the critical nucleation radius. The nucleation barrier can be further lowered by the presence of favorably interacting surfaces or nucleation sites in cases where droplet condensation occurs at defined nuclear locations.^95^ Indeed, some nuclear bodies are formed at specific genomic loci or structures; e.g., nucleoli form at rDNA,^96^ DNA damage foci form at PARylated regions,^97^ and transcriptional condensates form at active enhancer-promoter pairs.^19^ While there is growing interest in the biophysics of condensate nucleation,^95,98,99^ the application of concepts from classical nucleation theory to intracellular phase transitions is still in its infancy.

Just as other aspects of condensate phase behavior are complicated by a diversity of biological components, intracellular mechanics likely plays a significant role in the energetics and dynamics of droplet formation, particularly in the nucleus. In order to grow a large droplet within an elastic matrix, energy is required to deform the matrix, resulting in an additional energetic cost to phase separation. As a result, there will generally be an elevated saturation concentration, a reduced nucleation rate, and, depending on the properties of the matrix, a thermodynamically preferred droplet size. This phenomenon has only recently gained attention in soft matter physics,^62,100^ and connections to biology are still emerging.

In the context of nuclear biophysics, it remains challenging to probe and perturb chromatin mechanics in living cells, although early experimental evidence has begun to link such mechanical parameters to biomolecular phase separation in the nucleus. For example, droplets formed using engineered optogenetic systems preferentially nucleate in areas of low chromatin density and exclude bulk chromatin;^50^ this chromatin exclusion is also seen in various endogenous condensates such as the nucleolus and nuclear speckles. These observations suggest that the structural heterogeneity of the nucleus plays a role in the spatiotemporal regulation of the formation of membraneless organelles. This intuition was further supported both theoretically^101^ and in a synthetic experimental system:^100^ if the matrix is heterogeneous, growth of droplets in less stiff regions is energetically favored.

Following the initial phase of nucleation and growth, the evolution of the size distribution of droplets is driven by the minimization of total droplet surface area. This occurs by one of two mechanisms: Brownian motion driven coalescence, wherein mesoscopic droplets diffuse, collide and merge,^102^ or by Ostwald ripening, wherein molecules diffuse from small droplets to large droplets due to a surface-proximal concentration gradient specified by the Gibbs–Thomson relation.^103^ In the presence of a locally stiff matrix, however, this behavior can be suppressed or inverted because displacement of the matrix incurs an energetic penalty. This has been observed in molecular dynamics simulations of a heterogeneously linked chromatin polymer, suggesting that the local environment of the nucleus may enable the specific localization of condensates.^104^ This paradigm, however, only applies for condensates whose constituents demix with their mechanical environment, localizing to chromatin-poor regions.

In reality, the interplay of nuclear condensates and the chromatin is more nuanced. Emerging theoretical work has more systematically considered the possible interactions between droplets and an elastic meshwork beyond simple exclusion, generating a putative state diagram in the coordinates of the elasto-capillary number, h, and the permeo-elastic number, p [Fig. 3(f)]. The elasto-capillary number compares the elastic and surface energy and is defined as $h=\frac{3\gamma }{r_{m}G}$, where γ is the surface tension, $r_{m}$ is the matrix pore size, and G is the shear modulus of the network. The permeo-elastic number, which compares the permeation and deformation energies of the matrix, is defined as $p=\frac{\sigma_{P}}{G}$, where $\sigma_{p}$ is the permeation stress of the network, which can be thought of as the energy penalty for contact between the filaments of the network and the droplet phase compared to the dilute phase.^105^

If the surface tension and permeation stress are both high compared to the cost of deforming the network, i.e., h and p are both large, and a purely network-excluding condensate forms, similar to the experimental observations with various synthetic and native endogenous condensates.^50^ However, if the values of p and h are modest (i.e., ∼1), additional behaviors are possible. If p>h, i.e., the cost of having large total condensate surface area is not high and condensates strongly disfavor wetting the network, microdroplets form below the mesh size of the network. The opposite occurs if the surface tension dominates over the permeation stress, i.e., h>p, such that condensates partly wet chromatin [Fig. 3(f)]. The extreme of such behavior is cases where p is negative and the droplet is attracted to the network, which may be true of heterochromatic droplets, which recruit and even compact local chromatin^56,57,59^ if the magnitude of p is large.

As with all models, it is important to recognize the limitations of this conceptual framework. In particular, a given condensate can exhibit highly sequence-specific, targeted DNA wetting behavior. For example, the h>p case may be thought of as representing seeding onto specific genomic loci, e.g., Pol2 transcriptional condensates^106,107^ and polycomb condensates,^56,57^ which has also been reproduced in engineered systems seeded at specific genomic sites.^50,108^ However, intrinsically ordered regions (IDRs) on these same chromatin-associated proteins could at the same time exclude non-targeted chromatin regions, thereby acting as a specific chromatin filter.^50^ Moreover, because chromatin compaction is directly related to bulk nuclear mechanics, p and h should depend on total condensate size/amount, in a manner that is not accounted for by this model [Fig. 3(f)]. Nonetheless, these two dimensionless parameters provide a convenient framework that allows us to begin understanding how different behaviors observed for elastically constrained condensates can be understood from fundamental physical parameters.

## IV. CONCLUSIONS

Phase separation provides a compelling model for the functional organization of biomolecules in the nucleus and elsewhere within living cells. In recent years, significant progress has been made to move beyond overly simplified and sometimes only qualitative models and toward considering the ramifications that the uniquely complex environment of the cell has on phase separation. In particular, the mechanochemical complexity of the intracellular environment gives rise to unexpected behavior and indeed potentially even new physics. This includes behaviors not predicted by the simplest two-phase liquid (i.e., binary Flory–Huggins) model, for example, the presence of many stably sized aspherical droplets or a composition-dependent saturation concentration. One particularly intriguing “intracellular complication” is the viscoelasticity of the cell. While both nuclear bodies and nuclear mechanics have been separately investigated for decades, the conceptual framework of nuclear bodies as phase separated condensates provides an opening for understanding their interplay with nuclear mechanics. The effects of the mechanics of the environment on phase-separated droplets has only recently begun to be appreciated in biology. However, much further work is needed, including methods to measure the physical parameters, such as surface tension, permeation stress, and elasticity, that theory predicts will govern the condensate behavior in cells.

A second and equally interesting complication is the role of intracellular non-equilibrium activity. Our current theoretical approaches linking mechanics with phase separation generally assume thermodynamic equilibrium. In reality, both condensates and nuclear mechanics are highly biologically regulated. Major questions remain as to how cells simultaneously utilize both phenomena: Do cells use condensates as sensors of mechanical stimuli, e.g., to alter the epigenetic state and stiffness of the nucleus in response to external forces? Can condensates also directly alter local nuclear viscoelasticity to respond to changing mechanical requirements? A wide range of condensates may play such roles, on multiple time and length scales, ranging from maintaining heterochromatin-mediated bulk nuclear mechanics to coordinating protection and repair in response to DNA damage, suggesting a set of intricate, multifaceted answers to these questions. We anticipate that untangling the complex interactions between the maintenance of nuclear mechanics, the assembly of condensates, and their active regulation and response to external stimuli will drive new insights in both biology and physics.

## Data Availability

Data sharing is not applicable to this article as no new data were created or analyzed.
